# Retinal Vascular Lesions in Patients with Nonalcoholic Fatty Liver Disease: A Systematic Review and Meta-Analysis

**DOI:** 10.3390/jpm13071148

**Published:** 2023-07-17

**Authors:** Myrsini Orfanidou, Charikleia Ntenti, Kleo Evripidou, Asimina Mataftsi, Antonis Goulas, Stergios A. Polyzos

**Affiliations:** 1 First Laboratory of Pharmacology, School of Medicine, Aristotle University of Thessaloniki, 54124 Thessaloniki, Greece; chntenti@gmail.com (C.N.); kleo_evripidou@hotmail.com (K.E.); agoulas@auth.gr (A.G.); 2 Second Department of Ophthalmology, School of Medicine, Aristotle University of Thessaloniki, 54124 Thessaloniki, Greece; amatafts@auth.gr

**Keywords:** diabetes, nonalcoholic fatty liver disease, retinopathy, type 1 diabetes mellitus, type 2 diabetes mellitus

## Abstract

**Background:** This systematic review and meta-analysis aimed to summarize and compare data on retinal vascular lesions between patients with nonalcoholic fatty liver disease (NAFLD) and individuals without the disease. **Methods:** Search was performed in PubMed, Scopus and Cochrane Library, complemented by handsearching (PROSPERO ID: CRD42022345558). Thirty-six studies comprising 24,985 individuals (12,387 NAFLD patients and 12,598 controls) were selected for the meta-analysis. **Results:** Apart from retinopathy, no study with a different type of retinal vascular lesion was retrieved. Overall, there was no significant difference in the presence of retinopathy in NAFLD patients compared to controls (Odds Ratio (OR) = 1.20; 95% Confidence Interval (CI): 0.91–1.59). Heterogeneity among studies was high (I^2^ = 93%; *p* < 0.00001), while Egger’s test revealed no publication bias (*p* = 0.60). However, subgroup analysis showed positive association between retinopathy and NAFLD in type 1 diabetes mellitus (T1DM) (OR = 2.35; 95% CI: 1.53–3.60), but not in type 2 diabetes mellitus patients. Meta-regression analysis exploring potential confounders revealed no significant association. **Conclusions:** The presence of retinopathy was not overall different between individuals with and without NAFLD; however, T1DM patients with NAFLD had higher rates of retinopathy compared to T1DM patients without NAFLD, a finding warranting further research to show whether NAFLD may predict retinopathy in T1DM patients.

## 1. Introduction

Nonalcoholic fatty liver disease (NAFLD) is considered one of the leading causes of chronic liver disease worldwide, affecting about 30% of the global general adult population [1]. The prevalence of NAFLD is higher in specific population groups, such as in type 2 diabetes mellitus (T2DM) patients, reported to be 68% in Europe [2]. The continuous rise of the prevalence of NAFLD in the setting of multifactorial pathogenesis and current absence of officially approved treatment is a matter of concern, since NAFLD may progress from simple steatosis (or nonalcoholic fatty liver) to nonalcoholic steatohepatitis (NASH), liver fibrosis, cirrhosis and hepatocellular carcinoma [3]. Importantly, NAFLD is considered to be a multisystem disease [4]. Early mortality has been reported in NAFLD patients compared to individuals without the disease, which is primarily attributed to cardiovascular disorders [4]. In the setting of NAFLD as a multisystem disease [4], several studies have been published on the potential association between NAFLD and both macrovascular and microvascular complications, including chronic kidney disease, distal or autonomic neuropathy and retinopathy, as summarized by Mantovani et al. [5]. However, clinical studies regarding the association between NAFLD and retinopathy have, to date, provided conflicting data [6].

Secondary retinal vascular lesions, including microaneurysms, retinal hemorrhages (shaped as dots, blots or flames), hard exudates, cotton wool spots, retinal venular abnormalities (venous beading and tortuosity), intraretinal microvascular abnormalities and neovascularization, are known to be causally related to pathological conditions, such as hypertension and T2DM [7,8], which are both closely associated with NAFLD [9]. In this regard, most studies have, to date, focused on the association of NAFLD with diabetic retinopathy (DR) in patients with T2DM, as indicated by the meta-analysis of Song et al. [10]. However, there are also studies on this association in patients with type 1 diabetes mellitus (T1DM) and few studies in non-diabetic populations [11,12,13]. 

The aim of this systematic review and meta-analysis of observational studies was to summarize and compare data regarding the presence of retinal vascular lesions between patients with NAFLD and individuals without NAFLD, the latter serving as control group. 

## 2. Materials and Methods

### 2.1. Literature Search

This systematic review and meta-analysis was conducted following the reporting guidelines of the Meta-analysis of Observational Studies in Epidemiology (MOOSE) statement [14], and its protocol had a priori been preregistered in the PROSPERO registry (ID: CRD42022345558).

Systematic search was performed by two independent researchers (MO and CN) in the PubMed, Scopus and Cochrane Library electronic databases, without publication date or language restrictions. Based on the research question, a query was created combining Medical Subject Heading (MeSH) terms with non-MeSH terms. The query used for the search in PubMed was the following: ((“Non-alcoholic Fatty Liver Disease”[Mesh]) OR (Non-alcoholic Fatty Liver Disease) OR (Nonalcoholic Fatty Liver Disease) OR (Nonalcoholic Fatty Liver) OR (Non-alcoholic Fatty Liver) OR NAFLD OR NAFL OR NASH OR (Nonalcoholic Steatohepatitis) OR (Non-alcoholic Steatohepatitis) OR (Metabolic Associated Fatty Liver Disease) OR (Metabolic Dysfunction Associated Fatty Liver Disease) OR MAFLD) AND (“Retinal Diseases”[Mesh] OR (Retinal Disease) OR Retinopathy OR (Retinal Vascular Lesions) OR (Retinal Vascular Disease) OR Microangiopathy OR (Microvascular Complications)). The same query was used in the other two databases after taking into consideration their search string requirements and applying minimal, necessary modifications. Based on the same query, automatic alerts were activated in the same three databases in order to obtain relevant articles which were published after the initial search (27 September 2022).

As an extension to the aforementioned literature search, handsearching/manual searching was performed in three major international Gastroenterology and Hepatology congresses and two major international Ophthalmology congresses for the period of 2013–2022: the European Association for the Study of the Liver (EASL), the American Association for the Study of Liver Diseases (AASLD), the Asian Pacific Association for the Study of the Liver (APASL), the World Ophthalmology Congress (WOC) and the European Society of Retina Specialists (EURETINA). Moreover, the reference lists of all the selected articles were reviewed so as to retrieve further potentially relevant articles.

### 2.2. Inclusion and Exclusion Criteria

Observational studies (i.e., cross-sectional, case-control and cohort studies) that provided comparative data on retinal vascular lesions in patients with NAFLD and individuals without NAFLD were considered eligible for this systematic review and meta-analysis. The inclusion criteria were the following: (i) examining the adult population (age >18 y); (ii) presence of confirmation of NAFLD with one of the following methods: hepatic histology following liver biopsy, abdominal ultrasonography (US), computed tomography (CT), magnetic resonance imaging (MRI), magnetic resonance spectrometry (MRS), or non-invasive indices of hepatic steatosis and/or fibrosis; (iii) confirmation of retinal vascular lesions with one of the following methods: fundoscopy, fundus photography, angiography, optical coherence tomography (OCT).

On the other hand, studies were excluded if (i) patients presented with other liver disease(s) (e.g., viral hepatitis, alcoholic fatty liver disease, autoimmune hepatitis, drug-induced liver injury, Wilson’s disease, alpha-1 antitrypsin deficiency, etc.) or presented with NAFLD coexistent with other liver disease(s); (ii) patient overlap in two or more studies was confirmed; (iii) additional data were required, but were not provided by the respective corresponding author; (iv) the type of article was animal or cell line study, review, editorial, guidelines, opinion, commentary, hypothesis, note, book chapter, case report, case series or letter to the editor. Letters to the editor, however, were considered as potentially eligible during the selection process when they provided relevant original data.

### 2.3. Data Extraction

All articles that were initially retrieved were imported and saved in an EndNote file (Clarivate Analytics, Philadelphia, PA, USA) to facilitate their handling. At the stage of screening, two independent investigators (MO and CN) initially removed the duplicates and then reviewed all remaining articles, mainly based on their title and abstract. Afterwards, at the stage of eligibility, the two investigators (MO and CN) independently evaluated all the full-text articles. Any study not fulfilling the prespecified inclusion or meeting any of the exclusion criteria was excluded. In cases of disagreement between the investigators, the supervisor (SAP), who is qualified in systematic review and meta-analysis, was involved and moderated the discussion until a consensus was reached. 

All selected studies were thoroughly reviewed and the following parameters were extracted: (i) general characteristics (first author’s surname, country of origin, year of publication, design of the study); (ii) basic characteristics of the compared groups (number per group, sex, age, body mass index (BMI), waist circumference (WC), rates of diabetes mellitus (DM) and arterial hypertension); (iii) method used for the confirmation of retinal vascular lesions; (iv) method used for the confirmation of NAFLD; (v) biochemical measurements (aspartate aminotransferase (AST), alanine aminotransferase (ALT) and gamma-glutamyltranspeptidase (GGT) concentrations); (vi) homeostasis model assessment IR (HOMA-IR) calculating insulin resistance (IR). The extracted data were collected in an Excel spreadsheet (Microsoft Corp., Redmond, WA, USA).

When the full-text articles and/or their supplementary files could not provide some of the relevant data or when clarifications were considered necessary, we communicated via e-mail with the corresponding and/or the first and/or the senior authors. In cases of no response or unwillingness to provide us with the required information, we tried to calculate the necessary variables by using standard formulas, as elsewhere described in detail [15,16]. If the missing data were crucial (e.g., rate of retinal vascular lesions per group) and their estimation was not feasible, the study was considered ineligible and therefore was excluded from the meta-analysis. Google translate (https://translate.google.gr) was used as a tool for studies published in languages other than English; we also double-checked this procedure, by asking the corresponding authors of the relevant studies to validate the retrieved data.

When patient overlap between two or more studies occurred, we included only the study with the largest sample. If the sample size was the same, we included only the study providing most of the required information.

### 2.4. Quality Assessment

The quality of each included study was assessed by two independent investigators (MO and KE) using the Newcastle–Ottawa Scale (NOS; Ottawa Hospital Research Institute, Ottawa, ON, Canada). According to NOS, study validity was evaluated across three domains: the selection of the study groups (maximum 4 points); their comparability (maximum 2 points) and the assessment of the outcome (maximum 3 points). As a result, each selected study was assessed on a scale from 0 (very poor) to 9 (high). The supervisor (SAP) guided the reviewers throughout this procedure. In cases of disagreement between them, the supervisor moderated the discussion until a consensus was reached.

### 2.5. Outcomes and Statistical Analysis

The main outcome of this meta-analysis was the Odds Ratio (OR) of retinal vascular lesions between NAFLD patients and controls. The statistical analysis was performed using Revman (Review Manager, version 5.4.1; Cochrane Collaboration, London, UK) and “R” (R for Windows, version 4.2.2; the R Foundation for statistical computing, Vienna, Austria) software. The random-effects model was chosen for initial data analysis due to the expected heterogeneity of the outcome. The heterogeneity within studies was evaluated using the I^2^ test, while the possibility of publication bias was evaluated with visual review of funnel plot asymmetry and Egger’s test. All the statistical tests used were two-sided and the Confidence Interval (CI) was set at 95%.

We performed subgroup analysis based on the type of diabetes, i.e., separately for studies of patients with T2DM and T1DM. Studies in non-diabetic or mixed (i.e., T2DM and T1DM) populations were excluded from this analysis. Subgroup analysis was also performed according to the main method used for the confirmation of retinal vascular lesions (i.e., fundoscopy or fundus photography). Respectively, studies that did not provide relevant information were excluded. Sensitivity analyses were performed based on the quality assessment after excluding studies with (i) NOS < 6; (ii) NOS < 7. Additionally, sensitivity analysis was performed after the exclusion of studies with missing data regarding the method of the confirmation of retinal vascular lesions. Univariate random-effects meta-regression analysis was finally used, taking into account as potentially confounding factors (i) age; (ii) female percentage; (iii) BMI; (iv) WC; (v) arterial hypertension; (vi) ALT; (vii) AST; (viii) GGT.

## 3. Results

### 3.1. Literature Search

**Figure 1:**
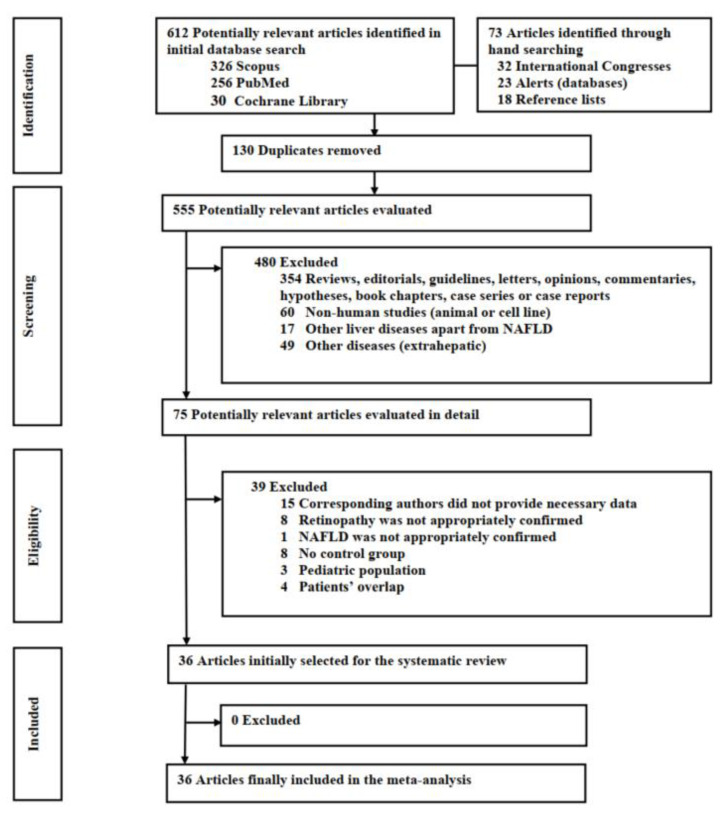
A flowchart summarizing the process of literature search according to the Preferred Reporting Items for Systematic reviews and Meta-Analyses (PRISMA) statement.

The initial database search retrieved 612 potentially relevant articles (326 from Scopus, 256 from PubMed and 30 from the Cochrane Library). A total of 73 additional articles were identified through handsearching (32 from International Congresses, 23 from database alerts and 18 from the references listed in the selected articles). Finally, 36 articles were included in the systematic review and the meta-analysis. A flowchart summarizing the stages of identification, screening, eligibility and inclusion of studies in this systematic review and meta-analysis, constructed according to the Preferred Reporting Items for Systematic reviews and Meta-Analyses (PRISMA) guidelines, is presented in Figure 1 [17]. It is noteworthy that, apart from retinopathy, no article with another vascular lesion as outcome was retrieved.

At the eligibility stage, we tried to communicate via e-mail with the corresponding and/or the first and/or the senior authors of 40 articles for which extra data were needed. The authors of 18 articles responded and provided us with all or part of the requested clarifications (one author provided us data from two included studies); their contribution is sincerely appreciated and recognized in the acknowledgment section. On the contrary, the authors of 22 articles did not respond or were unwilling to provide us with all necessary additional data or clarifications. As a result, 15 articles were excluded, because the required data were regarded as necessary (e.g., rate of retinopathy per group) and they were either not reported or their estimation was not feasible.

### 3.2. Characteristics of the Included Studies

The 36 selected studies were published between 2008 and 2023 and displayed data from 24,985 individuals (12,387 NAFLD patients and 12,598 controls) [11,12,13,18,19,20,21,22,23,24,25,26,27,28,29,30,31,32,33,34,35,36,37,38,39,40,41,42,43,44,45,46,47,48,49,50]. The main characteristics of the selected studies are presented in Table 1. Of those, 20 were carried out in Asia [18,20,21,22,23,26,28,30,35,37,38,39,40,44,45,46,47,48,49,50], 14 in Europe [11,12,19,24,25,29,31,32,33,34,36,41,42,43] and 2 in America [13,27]. A total of 30 were reported as cross-sectional [11,12,13,19,20,22,23,24,25,26,27,28,29,30,31,32,33,34,37,38,39,41,42,43,44,46,47,48,49,50], 4 as case-control [18,35,36,45] and 2 as cohort studies [21,40]. A total of 32 were performed on patients with DM [11,12,18,19,20,21,22,23,24,25,26,27,29,30,31,32,33,34,35,37,38,39,40,41,42,43,44,45,46,47,49,50]; 23 included only patients with T2DM [18,19,20,21,22,23,24,26,27,29,30,31,32,33,34,38,39,41,45,46,47,50], 6 included only patients with T1DM [11,12,42,43,44,49] and 2 reported on a mixed diabetic population [25,40], while in 1 article, the type of diabetes was not clarified [37]. Only one study stated clearly that the selected population consisted of patients without diabetes and hypertension [13]. Abdominal US was the prevailing method used for the diagnosis of NAFLD; 28 articles reported using abdominal US either solely or in combination with transient elastography for NAFLD confirmation [11,12,13,18,21,22,24,25,26,27,28,29,30,31,32,33,34,38,39,41,42,44,45,46,47,48,49,50]. At the same time, retinopathy was diagnosed mainly through fundoscopy (19 studies) [11,19,23,25,27,29,30,32,33,34,38,39,40,41,42,44,45,47,48], whereas 11 studies reported using fundus photography [12,13,18,22,24,26,28,36,43,46,50] and 6 studies did not provide relevant data [20,21,31,35,37,49]. All studies included both males and females between 25 and 83 years old, with the exception of one study that included only post-menopausal women [33]. The BMI ranged from normal to obese and only one study reported that only obese patients were recruited (Table S1) [21]. In addition, the rate of arterial hypertensive individuals was considerable when it was available, with the exception of only one study that included individuals without arterial hypertension [13]. *Vice versa*, there was also one study that included only hypertensive, diabetic patients (Table S1) [31]. The main demographic and laboratory characteristics of each study included in the systematic review and meta-analysis are presented in detail in Table S1.

### 3.3. Quality of the Included Studies

The Newcastle–Ottawa Scale was used to assess the quality of each selected study (Table 1 and  Table S2). The NOS score was four in one study, five in seven studies, six in twenty-one studies and seven in six studies. The mean (±standard deviation (SD)) NOS score was 5.91 ± 0.69. For one study (Conference abstract) [37], the NOS score was not estimated due to the lack of necessary information.

### 3.4. Outcomes of the Meta-Analysis

**Figure 2:**
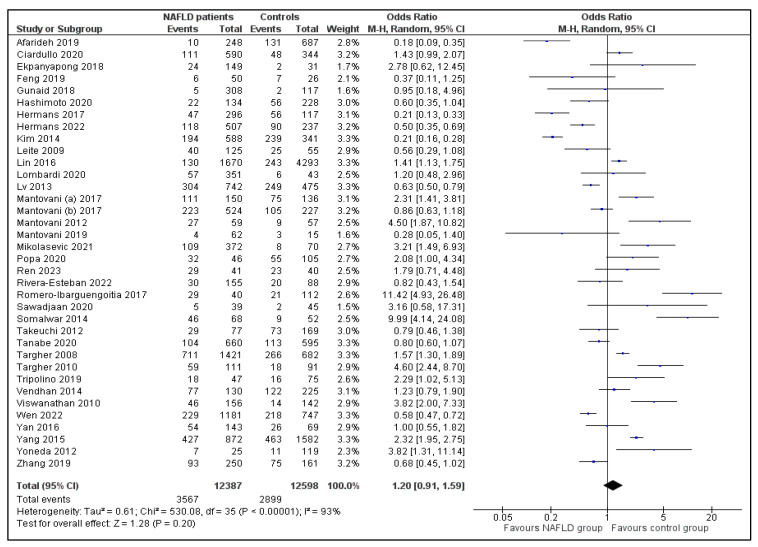
Forest plot displaying the quantitative synthesis of the Odds Ratio of retinopathy for the comparison between NAFLD patients and controls [11,12,13,18,19,20,21,22,23,24,25,26,27,28,29,30,31,32,33,34,35,36,37,38,39,40,41,42,43,44,45,46,47,48,49,50]. Data results from all studies (n = 36) included in the meta-analysis. Abbreviations: NAFLD, nonalcoholic fatty liver disease; M-H, Mantel–Haenszel; CI, Confidence Interval.

**Figure 3:**
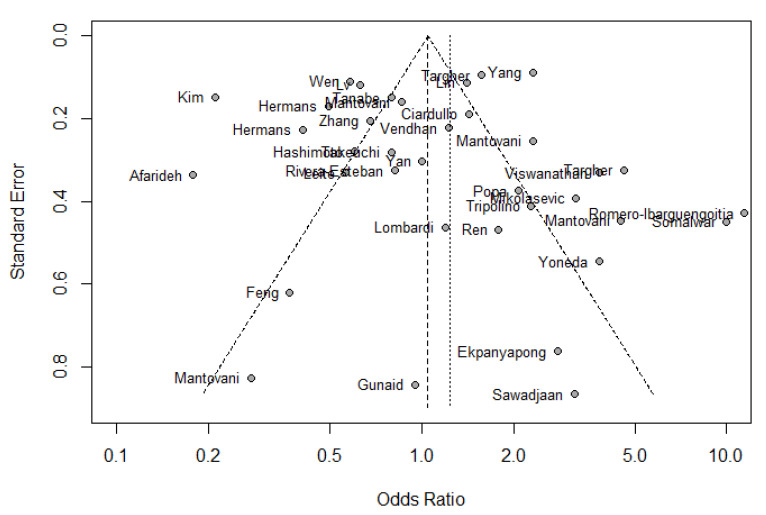
Funnel plot of the Odds Ratio of retinopathy for the comparison between NAFLD patients and controls [11,12,13,18,19,20,21,22,23,24,25,26,27,28,29,30,31,32,33,34,35,36,37,38,39,40,41,42,43,44,45,46,47,48,49,50].

As presented in Figure 2, the meta-analysis of the 36 selected studies indicated that the presence of retinopathy in patients with NAFLD was not significantly different compared to that in individuals without NAFLD (OR = 1.20; 95% CI (0.91, 1.59)). According to visual review of the funnel plot and Egger’s test, no significant publication bias was observed (*p* = 0.60; Figure 3 and Table 2). However, the heterogeneity among studies was high (I^2^ = 93%; *p* < 0.00001; Figure 2 and Table 2). In an attempt to interpret the heterogeneity, we performed subgroup, sensitivity and meta-regression analyses.

**Figure 4:**
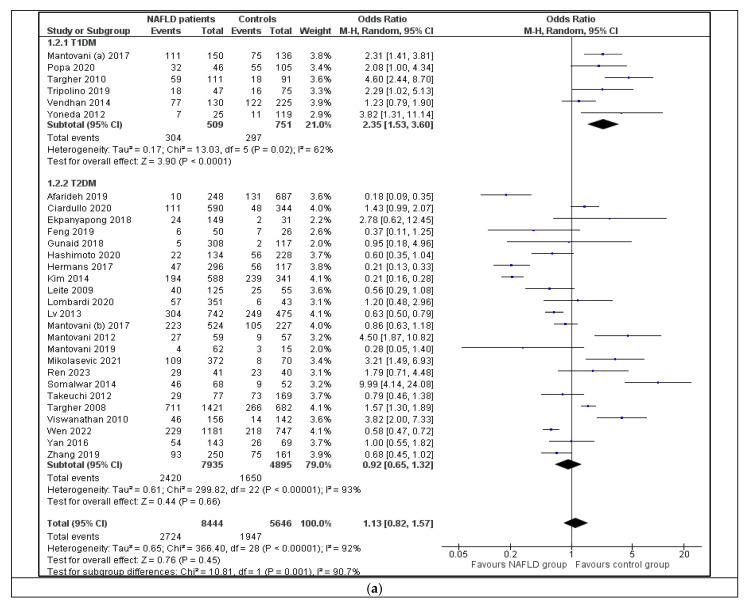

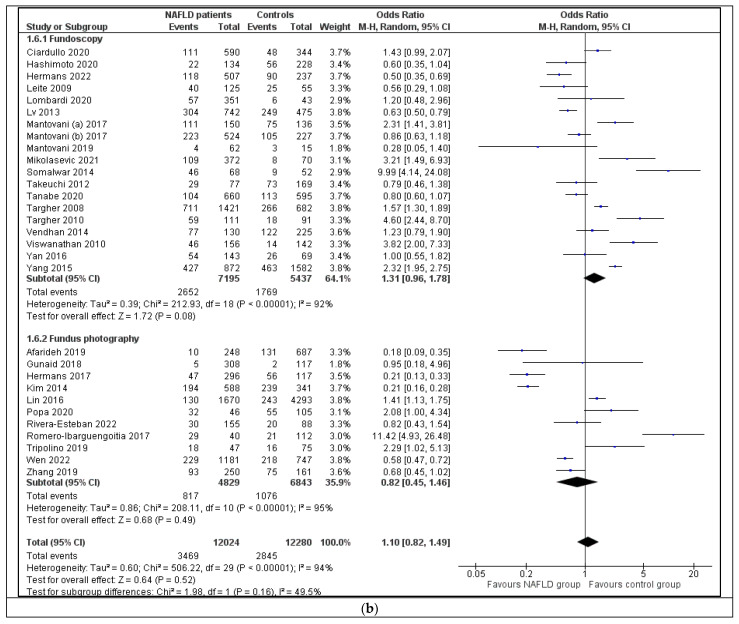
Forest plots displaying the quantitative synthesis of the Odds Ratio of retinopathy for the comparison between NAFLD patients and controls for subgroups classified according to (**a**) the type of diabetes (T1DM and T2DM) [11,12,18,19,20,21,22,23,24,26,27,29,30,31,32,33,34,35,38,39,41,42,43,44,45,46,47,49,50]; (**b**) the main method used for the diagnosis of retinopathy (fundoscopy and fundus photography) [11,12,13,18,19,22,23,24,25,26,27,28,29,30,32,33,34,36,38,39,40,41,42,43,44,45,46,47,48,50]. *Abbreviations:* NAFLD, nonalcoholic fatty liver disease; M-H, Mantel–Haenszel; CI, Confidence Interval; T1DM, type 1 diabetes mellitus; T2DM, type 2 diabetes mellitus.

The first subgroup analysis was performed based on the type of diabetes; the studies were classified into those including only patients with T1DM (n = 6) and those including only patients with T2DM (n = 23). Studies with individuals without DM, as well as those with definitely or probably mixed T1DM and T2DM population, were excluded (Table 1). This analysis resulted in statistically significant difference between subgroups (Figure 4a). More specifically, within the T2DM subgroup, the overall result was not statistically significant (OR = 0.92; 95% CI (0.65, 1.32)), i.e., the odds of retinopathy were not significantly different between patients with NAFLD and controls. The heterogeneity among studies remained high (I^2^ = 93%; *p* < 0.00001), while Egger’s test revealed no publication bias (*p* = 0.52; Figure S1a_1_). On the other hand, within the T1DM subgroup, the analysis showed a positive association between retinopathy and NAFLD [OR = 2.35; 95% CI (1.53, 3.60)]; i.e., patients with T1DM and NAFLD presented higher odds of retinopathy compared to individuals without NAFLD. The heterogeneity among studies remained statistically significant (I^2^ = 62%; *p* = 0.02), albeit lower than that within the T2DM subgroup (*p* = 0.001 for differences between subgroups); Egger’s test revealed no publication bias (*p* = 0.21; Figure S1a_2_).

Another subgroup analysis was performed based on the main method that was used for the diagnosis of retinopathy. Studies were divided into those that used fundoscopy (n = 19) and those that used fundus photography (n = 11). Six studies were excluded from the analysis due to data unavailability (Table 1). As displayed in Figure 4b, this analysis did not result in significant differences between subgroups (*p* = 0.160). Heterogeneity remained high within both groups (I^2^ = 92%, *p* < 0.00001; I^2^ = 95%, *p* < 0.00001, respectively), while Egger’s test revealed no publication bias (*p* = 0.87 and *p* = 0.60, respectively; Figure S1(b1,b2)).

Next, sensitivity analyses were performed after the exclusion of studies with (1) NOS < 6 (n = 9); (2) NOS < 7 (n = 30) and 3) unavailable data concerning the method of the diagnosis of retinopathy (n = 6; Table 1). The difference between groups regarding the risk of retinopathy remained non-significant in all these analyses (Figure S2). Heterogeneity among studies also remained high in all the analyses (I^2^ = 94%, *p* < 0.00001; I^2^ = 95%, *p* < 0.00001; I^2^ = 94%, *p* < 0.00001, respectively), and Egger’s test revealed no significant publication bias (*p* = 0.64, *p* = 0.91, *p* = 0.90, respectively; Table 2; Figure S1c–e).

Finally, meta-regression analysis was performed in order to explore the effect of potential confounding variables on the association between NAFLD and retinopathy. Probable confounders investigated included age, percentage of females, BMI, ALT, AST, GGT, WC and arterial hypertension. However, no significant association was observed, i.e., heterogeneity among studies could not be partially explained by any of these parameters (Table 3). Notably, arterial hypertension marginally failed to provide a significant association (*p* = 0.06; Table 3).

## 4. Discussion

This systematic review and meta-analysis of 36 observational studies showed overall similar rates of retinopathy between patients with NAFLD and individuals without NAFLD (Figure 2). However, within the subgroup of patients with T1DM, NAFLD patients had higher odds of retinopathy (OR = 2.35; 95% CI (1.53, 3.60)) compared to non-NAFLD controls, a finding that was not observed in the subgroup of patients with T2DM (Figure 4). Other subgroup, sensitivity and meta-regression analyses did not reveal significant differences between groups, and largely failed to interpret the high heterogeneity among studies of this systematic review and meta-analysis. Notably, publication bias was not observed in any of the comparisons performed during the study.

There are two previous meta-analyses available on this topic, published in 2021 [10] and 2022 [51] and including nine and eighteen studies, respectively. The former contained data on 7170 individuals, including only T2DM patients, and the latter included data on 12,757 individuals, including both T1DM and T2DM patients. Both studies showed similar results with our meta-analysis regarding T2DM [10,51], while the latter [51] showed higher rates of retinopathy in patients with T1DM and NAFLD compared to those with T1DM without NAFLD, in accordance with our results. The same study [51] also showed that NAFLD patients with liver fibrosis had higher rates of retinopathy compared to individuals without fibrosis; however, this finding should be cautiously interpreted, because fibrosis was non-invasively evaluated. More specifically, in the five studies included in that comparison [51], diagnosis of liver fibrosis was estimated either with NAFLD fibrosis score or liver stiffness upon transient elastography, i.e., with no histological data from biopsy-proven NAFLD populations. Taking the above into consideration, our systematic review and meta-analysis may be confirmatory of the finding of the two previous meta-analyses, but it has included an overall population that is about four [10] and two [51] times, respectively, larger than that of the previous two meta-analyses, i.e., implying more robust results due to a larger sample power. Importantly, the higher number of studies included in our meta-analysis compared to the previous meta-analyses is partly attributed to the handsearching we performed, which is not reported in the previous meta-analyses [10,51], implying potential selection bias.

Most of the studies included in our meta-analysis were performed in patients with DM, although our protocol did not prespecify such a limitation. This is not an unexpected finding, since it has been suggested that common pathogenic mechanisms underlie retinopathy and either DM or NAFLD, including IR, oxidative stress and inflammatory complications caused by impaired glucose and lipid metabolism [52,53,54]. Although any conclusions regarding the possible association between DR and NAFLD, drawn by both clinical observational studies and experimental studies, remain controversial, a variety of potential molecular mediators have been proposed to associate the eye with the liver, regulating communication and disease progression, such as advanced glycation end products, reactive oxygen species, C-reactive protein, interleukin-6, tumor necrosis factor-a, fibroblast growth factor-21 and hepatocyte growth factor [6]. However, more mechanistic studies are required based on all those potential mediators to show whether a causative effect of NAFLD on retinopathy exists and whether this is additive to that of DM. Following the results of this meta-analysis, this is considered of interest especially for models of T1DM. The co-existence of arterial hypertension, an established factor affecting retinopathy in DM, did not affect the association between NAFLD and retinopathy in this study, but, owing to the marginally non-statistical result of this meta-regression analysis, the potentially additive effect of arterial hypertension may warrant further research (Table 3).

Based on the design of this systematic review and meta-analysis, we could not draw any secure conclusions on the different patterns of the association between NAFLD and retinopathy in T1DM and T2DM, which needs, as already mentioned, mechanistic studies. This might have been attributed, at least partly, to an uneven covariate distribution (23 studies with T2DM vs. 6 with T1DM; Figure 4a), reflecting the higher prevalence of T2DM compared with T1DM in the general population. Furthermore, the distinct pathophysiology of T1DM and T2DM, as well as the more prolonged effect of the former compared to the latter type, may also be partly responsible for this observed difference. The higher oxidative stress, earlier exogenous insulin administration, as well as poorer glycemic control usually observed in patients with T1DM may further contribute to intrahepatic fat homeostasis disorder [55]. It is of note that the pathophysiology of T1DM, which is not characterized by IR at its early stages, is further complicated when IR is added, mainly, but not exclusively, as a consequence of central obesity observed in later stages of patients with T1DM on insulin treatment [56,57,58,59]. It is also noteworthy that the only study performed in patients without DM and arterial hypertension reported a significant association between NAFLD and retinopathy, implying that this association exists independently from DM and arterial hypertension [13]; however, other studies are needed to confirm this observation, especially given that the two previously published meta-analyses [10,51] as well as this meta-analysis showed no association between NAFLD and retinopathy overall.

This meta-analysis has two implications for future studies. First, NAFLD does not seem to be associated with retinopathy in patients with T2DM. Hence, it is questionable whether further relevant research is required, at least in terms of observational clinical studies in T2DM, which may be time- and resource-saving. Nevertheless, possible large studies of better design than the existing ones may provide results with less uncertainty, given the high heterogeneity within studies shown in this meta-analysis. On the other hand, the association between NAFLD and retinopathy in T1DM may warrant further research in terms of clinical studies, beyond the above-mentioned mechanistic studies. More specifically, cohort studies, ideally prospective ones, may be carried out to show whether NAFLD could serve as an early predictor of retinopathy in patients with T1DM. This would have been very important, because the early diagnosis of retinopathy, especially at its early stages, is a diagnostic asset for both patients and health systems.

This meta-analysis has some limitations: (i) All the included studies were observational, i.e., they cannot prove that an observed association reflects causation. Therefore, a cause–effect association between NAFLD and retinopathy cannot be supported. (ii) Heterogeneity among studies was high in most comparisons, and though further analyses (subgroup, sensitivity and meta-regression) were performed, the source(s) of the heterogeneity could not be partially explained; thus, the results of this meta-analysis should be cautiously interpreted. (iii) Although subgroup analysis based on the type of DM resulted in statistically significant difference between the comparison in T1DM and T2DM, these results should be cautiously interpreted because of the higher number of studies and patients with T2DM compared to those with T1DM (Figure 4a). (iv) The quality of the included studies was between four and seven based on NOS; thus, there were no studies of very low quality, and also there were no studies of very high quality among those selected for this meta-analysis. (v) Although the initial aim was not to include only retinopathy, no study with other retinal vascular lesions was retrieved as eligible for this systematic review and meta-analysis. (vi) Abdominal US was used to diagnose NAFLD in most of the included studies. We retrieved no data on histological confirmation of NAFLD, which is still considered to be the gold standard for the diagnosis and staging of the disease [60]. As a consequence, we did not perform comparisons between subgroups with and without histological confirmation or any analysis based on the histological classification of the disease (e.g., NAFL vs. NASH, liver fibrosis vs. no fibrosis, etc.). (vii) We did not perform meta-regression for HOMA-IR, which might have been important, because we retrieved HOMA-IR for only six studies (16.6%); this high rate of missing values might have impacted the result of this analysis. (viii) Apart from the quality assessment of the included studies with NOS, we did not perform an assessment based on Grading of Recommendations, Assessment, Development, and Evaluations (GRADE), because this was not a priori specified in the protocol of this meta-analysis as it was preregistered in the PROSPERO.

In conclusion, this systematic review and meta-analysis showed that retinopathy was overall not associated with NAFLD, with the exception of patients with T1DM, in whom NAFLD was associated with retinopathy. This finding warrants further mechanistic research to explore potential mediators of this association and to elucidate the underlying mechanisms, specifically in models of T1DM. Furthermore, cohort studies are needed to evaluate whether NAFLD could serve as an early predictor of retinopathy in patients with T1DM, a topic with highly valuable clinical implications.

## Figures and Tables

**Table 1:** Main characteristics of studies included in the systematic review and meta-analysis.

First Author’s Surname Reference ^†^	Year	Country	Study Design	Method of NAFLD Diagnosis	Method of Retinopathy Diagnosis	NOS Score	Additional Information
Afarideh [18]	2019	Iran	Case-control	Abdominal US	Fundus photography	5	Patients with T2DM
Ciardullo [19]	2020	Italy	Cross-sectional	Non-invasive indices	Fundoscopy	6	Patients with T2DM
Ekpanyapong [20]	2018	Thailand	Cross-sectional	Transient elastography	na	6	Patients with T2DM
Feng [21]	2019	China	Retrospective cohort	Abdominal US	na	4	Obese patients with T2DM
Gunaid [22]	2018	Yemen	Cross-sectional	Abdominal US	Fundus photography	6	Patients with T2DM
Hashimoto [23]	2020	Japan	Cross-sectional	Non-invasive indices	Fundoscopy	6	Patients with T2DM
Hermans [24]	2017	Belgium	Cross-sectional	Abdominal US	Fundus photography and/or angiography	6	Patients with T2DM
Hermans [25]	2022	Belgium	Cross-sectional	Abdominal US	Fundoscopy and/or angiography	7	Patients with diabetes
Kim [26]	2014	Korea	Cross-sectional	Abdominal US	Fundus photography and/or angiography	6	Patients with T2DM
Leite [27]	2009	Brazil	Cross-sectional	Abdominal US	Fundoscopy	6	Patients with T2DM
Lin [28]	2016	Taiwan	Cross-sectional	Abdominal US	Fundus photography	7	
Lombardi [29]	2020	Italy	Cross-sectional	Abdominal US and transient elastography	Fundoscopy	6	Patients with T2DM
Lv [30]	2013	China	Cross-sectional	Abdominal US	Fundoscopy	5	Patients with T2DM
Mantovani [31]	2012	Italy	Cross-sectional	Abdominal US	na	6	Patients with T2DM and hypertension
Mantovani (a) [11]	2017	Italy	Cross-sectional	Abdominal US	Fundoscopy and/or angiography	5	Patients with T1DM
Mantovani (b) [32]	2017	Italy	Cross-sectional	Abdominal US	Fundoscopy	6	Patients with T2DM
Mantovani [33]	2019	Italy	Cross-sectional	Abdominal US and transient elastography	Fundoscopy	7	Post-menopausal women with T2DM
Mikolasevic [34]	2021	Croatia	Cross-sectional	Abdominal US and transient elastography	Fundoscopy	6	Patients with T2DM
Popa [12]	2020	Romania	Cross-sectional	Abdominal US	Fundus photography	6	Patients with T1DM
Ren [35]	2023	China	Case-control	Abdominal MRI	na	6	Patients with T2DM
Rivera-Esteban [36]	2022	Spain	Case-control	Transient elastography	Fundus photography	5	
Romero-Ibarguengoitia [13]	2017	USA	Cross-sectional	Abdominal US	Fundus photography	6	Patients without diabetes and hypertension
Sawadjaan [37]	2020	Philippines	Cross-sectional	Transient elastography	na	na	Conference abstract; Patients with diabetes
Somalwar [38]	2014	India	Cross-sectional	Abdominal US	Fundoscopy	7	Patients with T2DM
Takeuchi [39]	2012	Japan	Cross-sectional	Abdominal US	Fundoscopy	6	Patients with T2DM
Tanabe [40]	2020	Japan	Retrospective cohort	Non-invasive indices	Fundoscopy	5	Patients with diabetes
Targher [41]	2008	Italy	Cross-sectional	Abdominal US	Fundoscopy and angiography	6	Patients with T2DM
Targher [42]	2010	Italy	Cross-sectional	Abdominal US	Fundoscopy and angiography	6	Patients with T1DM
Tripolino [43]	2019	Italy	Cross-sectional	Non-invasive indices	Fundus photography	6	Patients with T1DM
Vendhan [44]	2014	India	Cross-sectional	Abdominal US	Fundoscopy	6	Patients with T1DM
Viswanathan [45]	2010	India	Case-control	Abdominal US	Fundoscopy	7	Patients with T2DM
Wen [46]	2022	China	Cross-sectional	Abdominal US	Fundus photography	6	Patients with T2DM
Yan [47]	2016	China	Cross-sectional	Abdominal US	Fundoscopy	5	Patients with T2DM
Yang [48]	2015	China	Cross-sectional	Abdominal US	Fundoscopy	7	
Yoneda [49]	2012	Japan	Cross-sectional	Abdominal US	na	5	Patients with T1DM
Zhang [50]	2019	China	Cross-sectional	Abdominal US	Fundus photography	6	Patients with T2DM

^†^ References are classified according to the first author’s surname. *Abbreviations:* MRI, magnetic resonance imaging; na, not available; NAFLD, nonalcoholic fatty liver disease; NOS, Newcastle–Ottawa Scale; T1DM, type 1 diabetes mellitus; T2DM, type 2 diabetes mellitus; US, ultrasonography.

**Table 2:** Sensitivity analysis showing the OR of retinopathy compared between groups after exclusion of studies with NOS < 6 (third column), studies with NOS < 7 (fourth column) and studies with missing data regarding the method that was used for the diagnosis of retinopathy (fifth column).

Comparison	All Studies	After Excluding Studies with NOS < 6	After Excluding Studies with NOS < 7	After Excluding Studies without Known Method of Retinopathy Diagnosis
**Patients with NAFLD vs. patients without NAFLD**	OR = 1.20 (0.91, 1.59); *p* = 0.20 I^2^ = 93%; *p* < 0.00001 Egger’s test: *p* = 0.60	OR = 1.32 (0.94, 1.85); *p* = 0.11 I^2^ = 94%; *p* < 0.00001 Egger’s test: *p* = 0.64	OR = 1.74 (0.91, 3.33); *p* = 0.10 I^2^ = 95%; *p* < 0.00001 Egger’s test: *p* = 0.91	OR = 1.10 (0.82, 1.49); *p* = 0.52 I^2^ = 94%; *p* < 0.00001 Egger’s test: *p* = 0.90

*Abbreviations:* NAFLD, nonalcoholic fatty liver disease; NOS, Newcastle–Ottawa Scale; OR, Odds Ratio.

**Table 3:** Meta-regression analysis results concerning the effect of potential confounding factors on the association of NAFLD with retinopathy.

Potential Confounder (n of Studies)		
Age (years) (31)	Beta (95% CI); *p*-value	2.44 (−1.56, 6.43); 0.23
	Adjusted R square (%)	0.00
Females (%) (30)	Beta (95% CI); *p*-value	0.48 (−0.65, 1.62); 0.41
	Adjusted R square (%)	0.00
BMI (kg/m^2^) (31)	Beta (95% CI); *p*-value	1.98 (−2.01, 5.97); 0.33
	Adjusted R square (%)	0.00
ALT (IU/L) (25)	Beta (95% CI); *p*-value	0.44 (−0.68, 1.56); 0.44
	Adjusted R square (%)	0.00
AST (IU/L) (24)	Beta (95% CI); *p*-value	−0.69 (−2.64, 1.27); 0.49
	Adjusted R square (%)	1.06
GGT (IU/L) (19)	Beta (95% CI); *p*-value	0.30 (−0.32, 0.93); 0.35
	Adjusted R square (%)	0.00
Waist circumference (cm) (16)	Beta (95% CI); *p*-value	4.86 (−2.52, 12.25); 0.20
	Adjusted R square (%)	1.87
Arterial hypertension (20)	Beta (95% CI); *p*-value	0.99 (−0.04, 2.01); 0.06
	Adjusted R square (%)	9.23

*Abbreviations:* ALT, alanine aminotransferase; AST, aspartate aminotransferase; BMI, Body Mass Index; CI, Confidence Interval; GGT, gamma-glutamyltranspeptidase; NAFLD, nonalcoholic fatty liver disease.

## Data Availability

All data used for this meta-analysis are presented in Table 1 and in Supplementary Table S1.

## Supplementary Materials

The following supporting information can be downloaded at: https://www.mdpi.com/article/10.3390/jpm13071148/s1, Figure S1: Funnel plot of the Odds Ratio of retinopathy for the comparison between NAFLD patients and controls for: (a1) the subgroup analysis based on the type of diabetes: subgroup of type 2 diabetes mellitus; (a2) the subgroup analysis based on the type of diabetes: subgroup of type 1 diabetes mellitus; (b1) the subgroup analysis based on the main method used for the diagnosis of retinopathy: subgroup of fundoscopy; (b2) the subgroup analysis based on the main method used for the diagnosis of retinopathy: subgroup of fundus photography; (c) the sensitivity analysis after exclusion of studies with NOS < 6; (d) the sensitivity analysis after exclusion of studies with NOS < 7; (e) the sensitivity analysis after exclusion of studies with not available data regarding the method used for the diagnosis of retinopathy.; Figure S2: Forest plots displaying the quantitative synthesis of the Odds Ratio of retinopathy for the comparison between NAFLD patients and controls after excluding studies with: (a) NOS < 6; (b) NOS < 7; (c) not available data regarding the method used for the diagnosis of retinopathy.; Table S1: The main demographic and laboratory characteristics per group of each study included in the systematic review and meta-analysis.; Table S2: Quality assessment of the studies included in the systematic review and meta-analysis.
