# Supplementary material for: Retinal Vascular Lesions in Patients with Nonalcoholic Fatty Liver Disease: A Systematic Review and Meta-Analysis
Source: J Pers Med. 2023 Jul 17;13(7):1148. doi: 10.3390/jpm13071148 (PMC10381395; doi:10.3390/jpm13071148)
Supplement: Supplementary file 1 [file jpm-13-01148-s001.zip › supplementary tables.pdf]

Table S1. The main demographic and laboratory characteristics per group of each study included in the systematic review and meta-analysis.

**First author, Year, Origin** **Reference** *	**Group**	**N**	**Females (n)**	**DM (n)**	**Hypertension (n)**	**Age (years)**	**BMI (kg/m2)**	**Waist circumference (cm)**	**AST (IU/L)**	**ALT (IU/L)**	**GGT (IU/L)**	**HOMA-IR**	**Retinopathy (n)**
Afarideh, 2019, Iran[18]	Control	687	485†	687	na	57.64 ± 9.38†	29.00 ± 4.71†	98.68 ± 10.43†	na	na	na	3.94 ± 2.76	131
	NAFLD	248		248	na				na	na	na		10
Ciardullo, 2020, Italy[19]	Control	344	242†	344	na	67.00 ± 11.00†	30.56 ± 6.00†	106.00 ± 14.00†	22.00 ± 14.00†	26.00 ± 20.00†	47.00 ± 70.00†	na	48
	NAFLD	590		590	na							na	111
Ekpanyapong, 2018, Thailand[20]	Control	31	118†	31	129†	58.50 ± 12.00†	26.80 ± 4.80†	90.10 ± 11.20†	20.40 ± 5.70†	20.70 ± 7.50†	na	na	2
	NAFLD	149		149							na	na	24
Feng, 2019, China[21]	Control	26	34†	26	45†	46.61 ± 3.35†	32.73 ± 0.90†	108.26 ± 1.80†	na	na	na	na	7

	NAFLD	50		50					na	na	na	na	6
Gunaid, 2018, Yemen[22]	Control	117	na	117	na	na	na	na	na	na	na	na	2
	NAFLD	308	na	308	na	na	na	na	na	na	na	na	5
Hashimoto, 2020, Japan[23]	Control	228	171†	228	na	66.40 ± 10.60†	24.30 ± 4.10†	na	24.00 ± 1.71†	20.00 ± 2.39†	na	na	56
	NAFLD	134		134	na			na			na	na	22
Hermans, 2017, Belgium[24]	Control	117	na	117	na	na	na	na	na	na	na	na	56
	NAFLD	296	na	296	na	na	na	na	na	na	na	na	47
Hermans, 2022, Belgium[25]	Control	237	102	237	206	70.00 ± 13.00†	29.10 ± 5.80†	103.00 ± 14.00†	27.00 ± 15.00†	30.00 ± 24.00†	47.00 ± 58.00†	na	90
	NAFLD	507	188	507	454							na	118
Kim, 2014, Korea[26]	Control	341	175	341	205	59.30 ± 11.90	23.30 ± 3.40	na	25.10 ± 43.70	30.10 ± 80.90	49.20 ± 52.50	3.30 ± 2.80	239

	NAFLD	588	264	588	406	56.70 ± 11.70	25.80 ± 3.50	na	29.20 ± 37.50	37.90 ± 68.80	67.50 ± 138.60	5.00 ± 4.80	194
Leite, 2009, Brazil[27]	Control	55	38	55	43	54.30 ± 8.10	27.00 ± 3.60	95.30 ± 7.70	21.5 ± 2.19	35.75 ± 1.97	36.25 ± 5.48	na	25
	NAFLD	125	88	125	106	55.60 ± 7.10	31.30 ± 5.40	104.60 ± 12.20	20.75 ± 1.75	40.00 ± 3.11	42.00 ± 5.82	na	40
Lin, 2016, Taiwan[28]	Control	4293	2418	486	na	54.03 ± 0.32[†]	na	95.74 ± 0.33[†]	na	na	na	na	243
	NAFLD	1670	784	459	na		na		na	na	na	na	130
Lombardi, 2020, Italy[29]	Control	43	189[†]	43	306[†]	68.00 ± 10.00[†]	29.00 ± 5.00[†]	101.00 ± 12.00[†]	39.50 ± 17.62[†]	73.75 ± 41.85[†]	182.25 ± 114.38[†]	na	6
	NAFLD	351		351								na	57
Lv, 2013, China[30]	Control	475	287	475	na	65.11 ± 11.70	24.33 ± 2.87	88.75 ± 8.72	18.23 ± 6.74	17.16 ± 8.19	19.47 ± 16.32	na	249
	NAFLD	742	470	742	na	62.29 ± 12.52	27.49 ± 3.37	96.76 ± 10.32	22.34 ± 9.00	25.72 ± 13.01	25.05 ± 17.94	na	304

Mantovani, 2012, Italy[31]	Control	57	60†	57	57	68.17 ± 13.54†	31.34 ± 8.06†	na	29.91 ± 19.86†	29.83 ± 25.90†	43.21 ± 36.37†	na	9
	NAFLD	59		59	59			na				na	27
Mantovani, 2017, Italy (a)[11]	Control	136	87	136	42	38.80 ± 13.10	22.70 ± 3.40	na	18.00 ± 2.30	18.50 ± 1.92	14.75 ± 2.11	na	75
	NAFLD	150	78	150	91	47.50 ± 14.20	26.10 ± 4.70	na	18.25 ± 2.08	21.25 ± 2.84	23.50 ± 4.93	na	111
Mantovani, 2017, Italy (b)[32]	Control	227	346†	227	na	na	30.43 ± 6.90†	na	na	22.58 ± 3.00†	33.91 ± 6.46†	na	105
	NAFLD	524		524	na	na		na	na			na	223
Mantovani, 2019, Italy[33]	Control	15	15	15	14	76.80 ± 7.00	27.10 ± 5.00	90.20 ± 11.00	21.00 ± 5.00	11.00 ± 3.00	12.75 ± 2.01	1.20 ± 0.34	3
	NAFLD	62	62	62	48	70.88 ± 7.29	29.6 ± 4.82	99.52 ± 12.53	23.13 ± 7.90	14.65 ± 7.42	21.07 ± 9.24	2.81 ± 1.22	4
Mikolasevic, 2021, Croatia[34]	Control	70	na	70	351†	61.00 ± 11.17	30.57 ± 5.21	106.60 ± 13.00	23.00 ± 6.69	26.33 ± 12.64	30.00 ± 20.08	na	8
	NAFLD	372	na	372								na	109

Popa, 2020, Romania[12]	Control	105	58	105	48	38.50 ± 13.00	23.40 ± 3.60	83.60 ± 10.90	20.00 ± 11.50	21.00 ± 14.00	18.00 ± 14.50	na	55
	NAFLD	46	16	46	24	44.90 ± 12.10	25.60 ± 4.50	93.80 ± 13.50	25.00 ± 17.00	26.00 ± 16.70	24.00 ± 22.50	na	32
Ren, 2023, China[35]	Control	40	21	40	na	56.90 ± 9.80	23.90 ± 3.90	na	19.20 ± 7.40	21.70 ± 14.70	28.90 ± 24.80	2.10 ± 1.00	23
	NAFLD	41	18	41	na	53.90 ± 10.60	26.20 ± 4.00	na	31.50 ± 26.10	49.90 ± 60.90	39.90 ± 30.20	3.40 ± 1.90	29
Rivera-Esteban, 2022, Spain[36]	Control	88	na	62	na	na	na	na	na	na	na	na	20
	NAFLD	155	na	124	na	na	na	na	na	na	na	na	30
Romero-Ibarguengoitia, 2017, USA[13]	Control	112	106[†]	0	0	28.36 ± 8.11	24.50 ± 5.10	na	22.54 ± 7.00	21.48 ± 13.50	na	na	21
	NAFLD	40		0	0	35.50 ± 7.46	35.50 ± 7.80	na	26.30 ± 1.12	32.90 ± 13.95	na	na	29
Sawadjaan, 2020, Philippines[37]	Control	45	35[†]	45	na	56.12 ± 13.04[†]	29.01 ± 5.88[†]	na	na	na	na	na	2

	NAFLD	39		39	na			na	na	na	na	na	5
Somalwar, 2014, India[38]	Control	52	69†	52	na	54.51 ± 5.46	22.98 ± 2.62	83.19 ± 5.01	22.07 ± 5.11	22.90 ± 6.03	na	na	9
	NAFLD	68		68	na	55.73 ± 6.08	26.97 ± 1.78	93.66 ± 6.66	35.79 ± 6.27	40.70 ± 7.06	na	na	46
Takeuchi, 2012, Japan[39]	Control	169	46†	169	70	65.00 ± 7.00	23.70 ± 3.70	na	30.00 ± 54.00†	29.00 ± 42.00†	44.00 ± 101.00†	na	73
	NAFLD	77		77	81	61.00 ± 9.00	26.40 ± 3.60	na				na	29
Tanabe, 2020, Japan[40]	Control	595	562†	595	949†	58.01 ± 3.88†	25.16 ± 1.78†	na	na	na	na	1.57 ± 0.60†	113
	NAFLD	660		660				na	na	na	na		104
Targher, 2008, Italy[41]	Control	682	280	682	na	57.00 ± 3.00	26.00 ± 4.00	92.00 ± 11.00	18.00 ± 4.00	20.00 ± 4.00	20.00 ± 5.00	na	266
	NAFLD	1421	526	1421	na	59.00 ± 4.00	27.00 ± 3.00	95.00 ± 13.00	23.00 ± 9.00	25.00 ± 11.00	27.00 ± 10.00	na	711

Targher, 2010, Italy[42]	Control	91	55	91	na	37.00 ± 12.00	22.50 ± 3.00	na	20.00 ± 6.00	20.00 ± 8.00	17.00 ± 9.00	na	18
	NAFLD	111	41	111	na	47.00 ± 12.00	26.60 ± 5.00	na	25.00 ± 11.00	29.00 ± 17.00	29.00 ± 20.00	na	59
Tripolino, 2019, Italy[43]	Control	75	54[†]	75	14[†]	37.00 ± 11.30[†]	24.57 ± 3.85[†]	na	na	na	na	na	16
	NAFLD	47		47				na	na	na	na	na	18
Vendhan, 2014, India[44]	Control	225	na	na	na	na	na	na	na	na	na	na	122
	NAFLD	130	na	na	na	na	na	na	na	na	na	na	77
Viswanathan, 2010, India[45]	Control	142	52	142	58	49.00 ± 11.00	26.40 ± 4.00	na	27.60 ± 17.30	22.40 ± 15.40	na	na	14
	NAFLD	156	55	156	101	50.00 ± 10.00	29.70 ± 7.00	na	37.60 ± 24.90	29.30 ± 17.90	na	na	46
Wen, 2022, China[46]	Control	747	364	747	393	60.00 ± 13.00	24.07 ± 3.15	89.30 ± 9.60	16.50 ± 0.95	14.75 ± 1.43	na	1.20 ± 0.13	218

	NAFLD	1181	548	1181	642	55.00 ± 14.00	27.31 ± 3.80	97.60 ± 10.50	19.75 ± 1.68	23.25 ± 2.90	na	1.83 ± 0.17	229
Yan, 2016, China[47]	Control	69	31	69	28	56.74 ± 11.51	23.75 ± 3.78	na	16.74 ± 5.08	17.20 ± 8.01	21.35 ± 18.75	na	26
	NAFLD	143	61	143	76	52.20 ± 10.07	28.41 ± 3.58	na	23.70 ± 17.76	20.23 ± 10.75	43.32 ± 34.54	na	54
Yang, 2015, China[48]	Control	1582	224	167	406	61.94 ± 9.95	24.31 ± 2.90	na	na	na	na	na	463
	NAFLD	872	87	114	304	63.06 ± 10.13	26.19 ± 3.14	na	na	na	na	na	427
Yoneda, 2012, Japan[49]	Control	119	65	119	na	47.90 ± 1.40	21.90 ± 0.20	na	20.80 ± 0.70	18.60 ± 0.90	26.50 ± 5.10	na	11
	NAFLD	25	17	25	na	54.00 ± 3.00	26.80 ± 0.60	na	26.70 ± 2.70	27.30 ± 3.40	63.20 ± 19.70	na	7
Zhang, 2019, China[50]	Control	161	70	161	32	58.04 ± 10.68	24.08 ± 3.03	88.80 ± 8.87	21.50 ± 1.88	20.12 ± 18.61	20.75 ± 2.63	na	75
	NAFLD	250	145	250	48	58.44 ± 11.80	26.69 ± 3.69	95.16 ± 11.80	24.00 ± 2.49	29.10 ± 3.35	27.50 ± 3.56	na	93

* References are classified according to the first author's surname.

† Data referring to the whole population instead of per group for each study.

Abbreviations: ALT, alanine aminotransaminase; AST, aspartate aminotransaminase; BMI, body mass index; DM, diabetes mellitus; GGT, gamma-glutamyltranspeptidase; na, not available; HOMA-IR, homeostasis model assessment insulin resistance.

Table S2. Quality assessment of the studies included in the systematic review and meta-analysis.

	SELECTION				COMPARABILITY			EXPOSURE		
First author, Year, Origin Reference *	Case Definition	Case Representativeness	Control Selection	Control Definition	BMI	AGE	Exposure	Exposure Ascertainment (cases v controls)	Non-response rate	NOS Score
Afarideh, 2019, Iran[18]	1	0	0	1	0	0	1	1	1	5
Ciardullo, 2020, Italy[19]	1	1	0	1	0	0	1	1	1	6
Ekpanyapong, 2018, Thailand[20]	1	1	1	1	0	0	0	1	1	6
Feng, 2019, China[21]	1	0	0	1	0	0	0	1	1	4
Gunaid, 2018, Yemen[22]	1	1	0	1	0	0	1	1	1	6
Hashimoto, 2020, Japan[23]	1	1	0	1	0	0	1	1	1	6
Hermans, 2017, Belgium[24]	1	1	0	1	0	0	1	1	1	6
Hermans, 2022, Belgium[25]	1	1	0	1	0	1	1	1	1	7
Kim, 2014, Korea[26]	1	1	0	1	0	0	1	1	1	6
Leite, 2009, Brazil[27]	1	0	0	1	0	1	1	1	1	6
Lin, 2016, Taiwan[28]	1	1	1	1	0	0	1	1	1	7
Lombardi, 2020, Italy[29]	1	1	0	1	0	0	1	1	1	6
Lv, 2013, China[30]	1	0	0	1	0	0	1	1	1	5
Mantovani, 2012, Italy[31]	1	1	0	1	0	0	0	1	1	5
Mantovani (a), 2017, Italy[11]	1	1	0	1	0	0	1	1	1	6
Mantovani (b), 2017, Italy[32]	1	1	0	1	0	0	1	1	1	6
Mantovani, 2019, Italy[33]	1	1	0	1	1	0	1	1	1	7
Mikolasevic, 2021, Croatia[34]	1	1	0	1	0	0	1	1	1	6
Popa, 2020, Romania[12]	1	1	0	1	0	0	1	1	1	6
Ren, 2023, China[35]	1	1	0	1	0	1	0	1	1	6
Rivera-Esteban, 2022, Spain[36]	1	0	0	1	0	0	1	1	1	5
Romero-Ibarguengoitia, 2017, USA[13]	1	1	0	1	0	0	1	1	1	6
Sawadjaan, 2020, Philippines[37]	na	na	na	na	na	na	na	na	na	na

Somalwar, 2014, India[38]	1	1	0	1	0	1	1	1	1	7
Takeuchi, 2012, Japan[39]	1	1	0	1	0	0	1	1	1	6
Tanabe, 2020, Japan[40]	1	0	0	1	0	0	1	1	1	5
Targher, 2008, Italy[41]	1	1	0	1	0	0	1	1	1	6
Targher, 2010, Italy[42]	1	1	0	1	0	0	1	1	1	6
Tripolino, 2019, Italy[43]	1	1	0	1	0	0	1	1	1	6
Vendhan, 2014, India[44]	1	1	0	1	0	0	1	1	1	6
Viswanathan, 2010, India[45]	1	1	0	1	0	1	1	1	1	7
Wen, 2022, China[46]	1	1	0	1	0	0	1	1	1	6
Yan, 2016, China[47]	1	0	0	1	0	0	1	1	1	5
Yang, 2015, China[48]	1	1	1	1	0	0	1	1	1	7
Yoneda, 2012, Japan[49]	1	0	0	1	0	1	0	1	1	5
Zhang, 2019, China[50]	1	0	0	1	0	1	1	1	1	6

[*] References are classified according to the first author's surname.

Abbreviations: BMI, body mass index; na, not available; NOS, Newcastle-Ottawa Scale
