# Supplementary figures and images for: Retinal Vascular Lesions in Patients with Nonalcoholic Fatty Liver Disease: A Systematic Review and Meta-Analysis
Source: J Pers Med. 2023 Jul 17;13(7):1148. doi: 10.3390/jpm13071148 (PMC10381395; doi:10.3390/jpm13071148)

## Slide 1
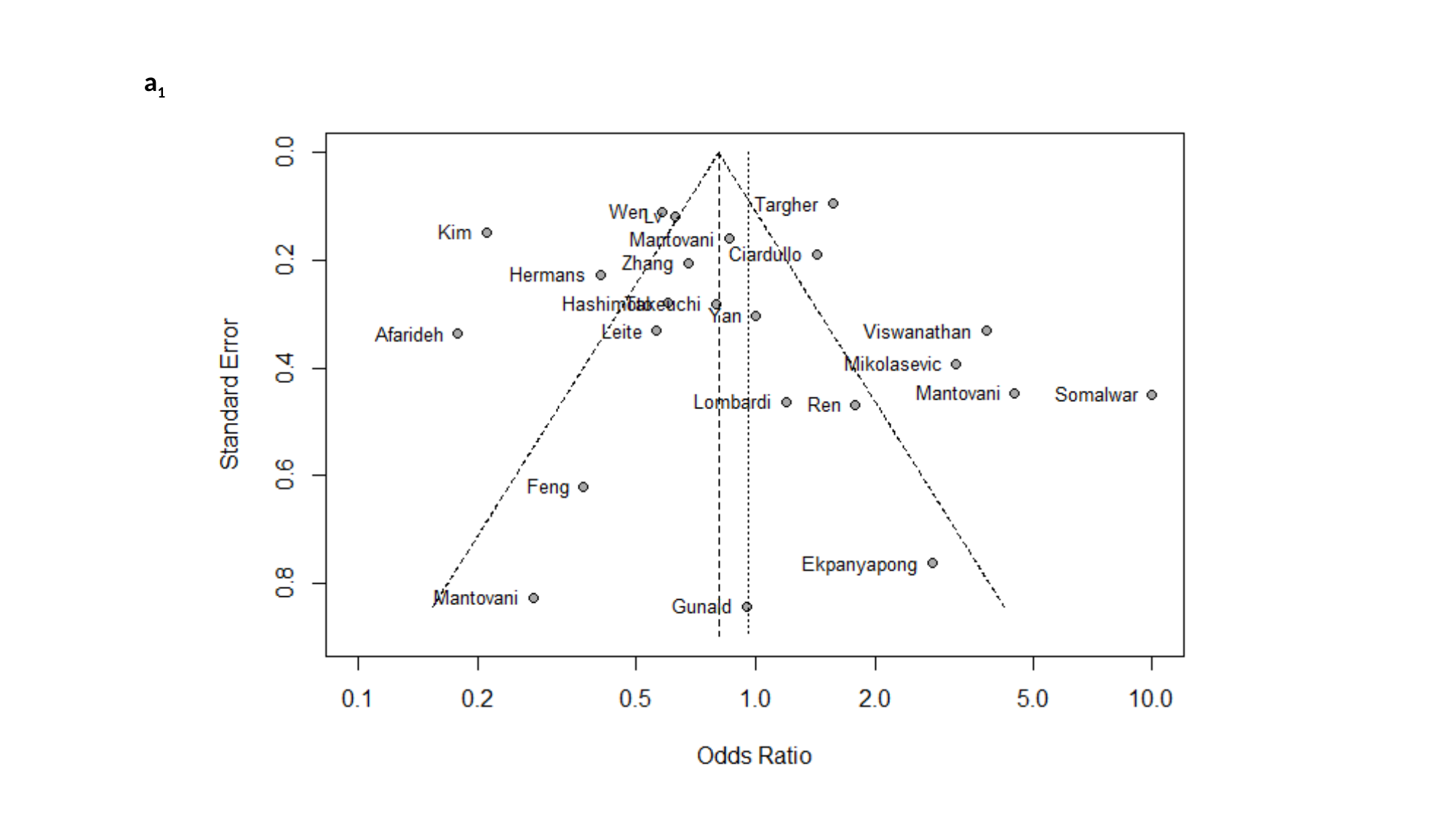

a1

## Slide 2
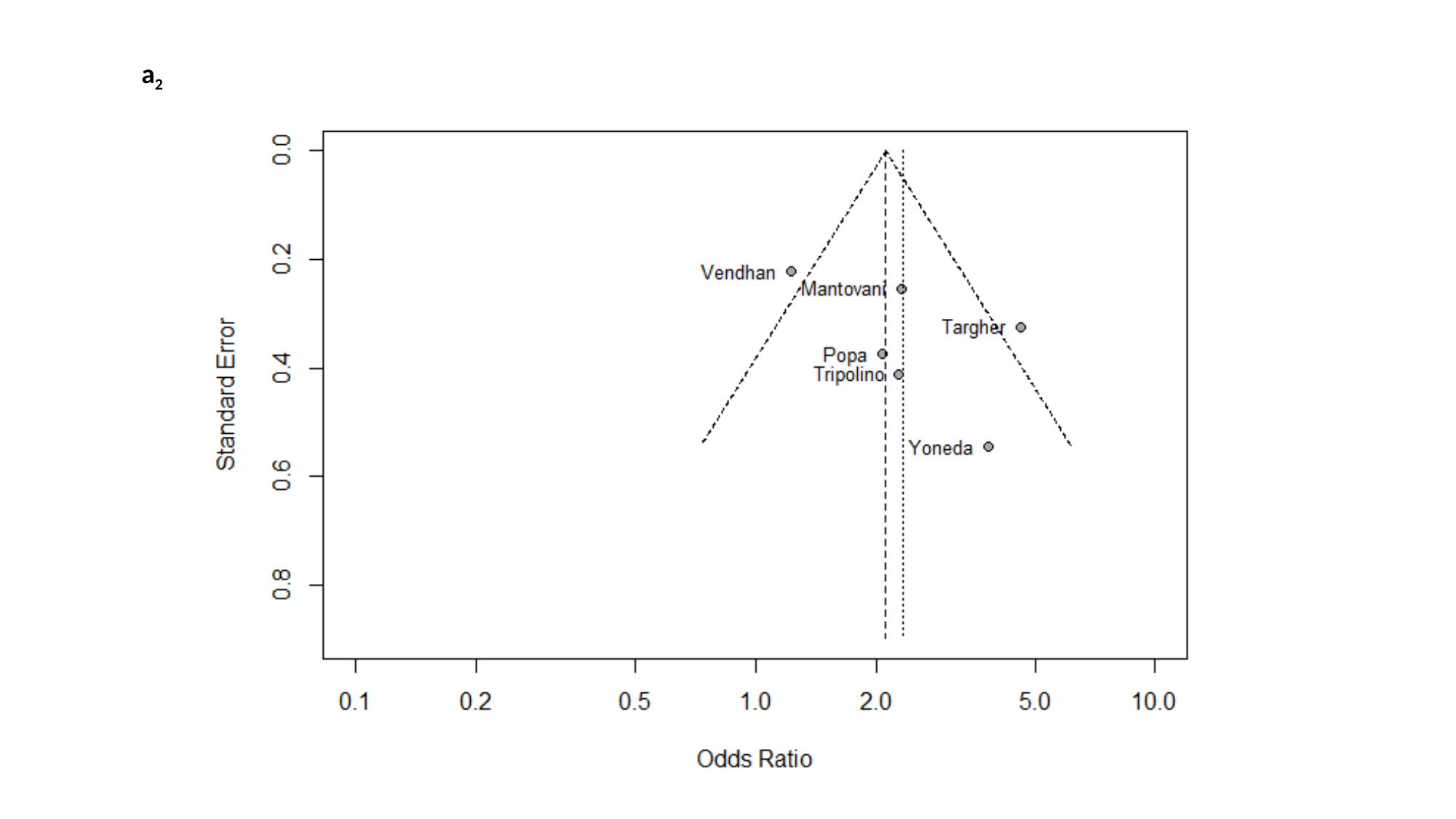

a2

## Slide 3
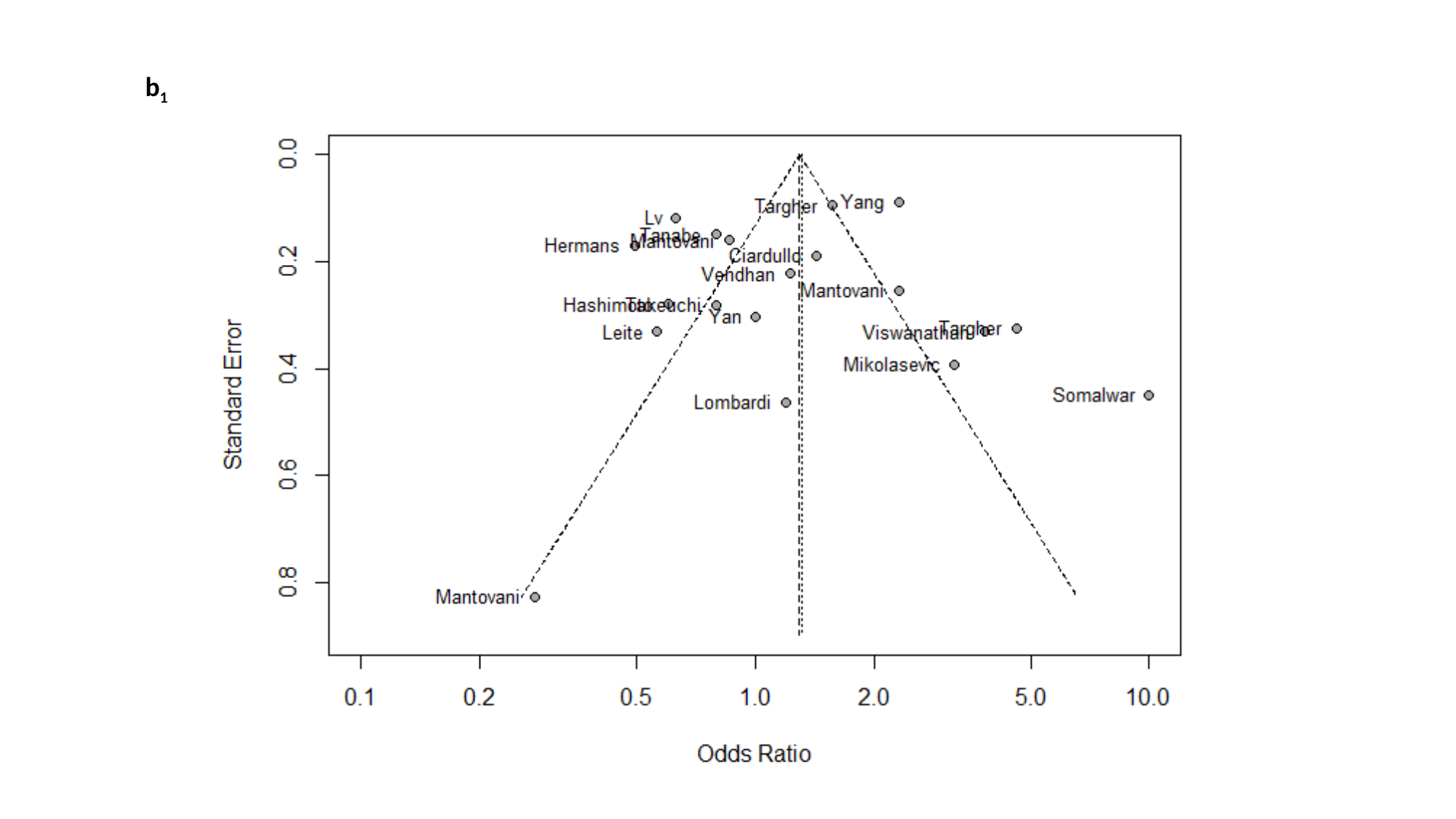

b1

## Slide 4
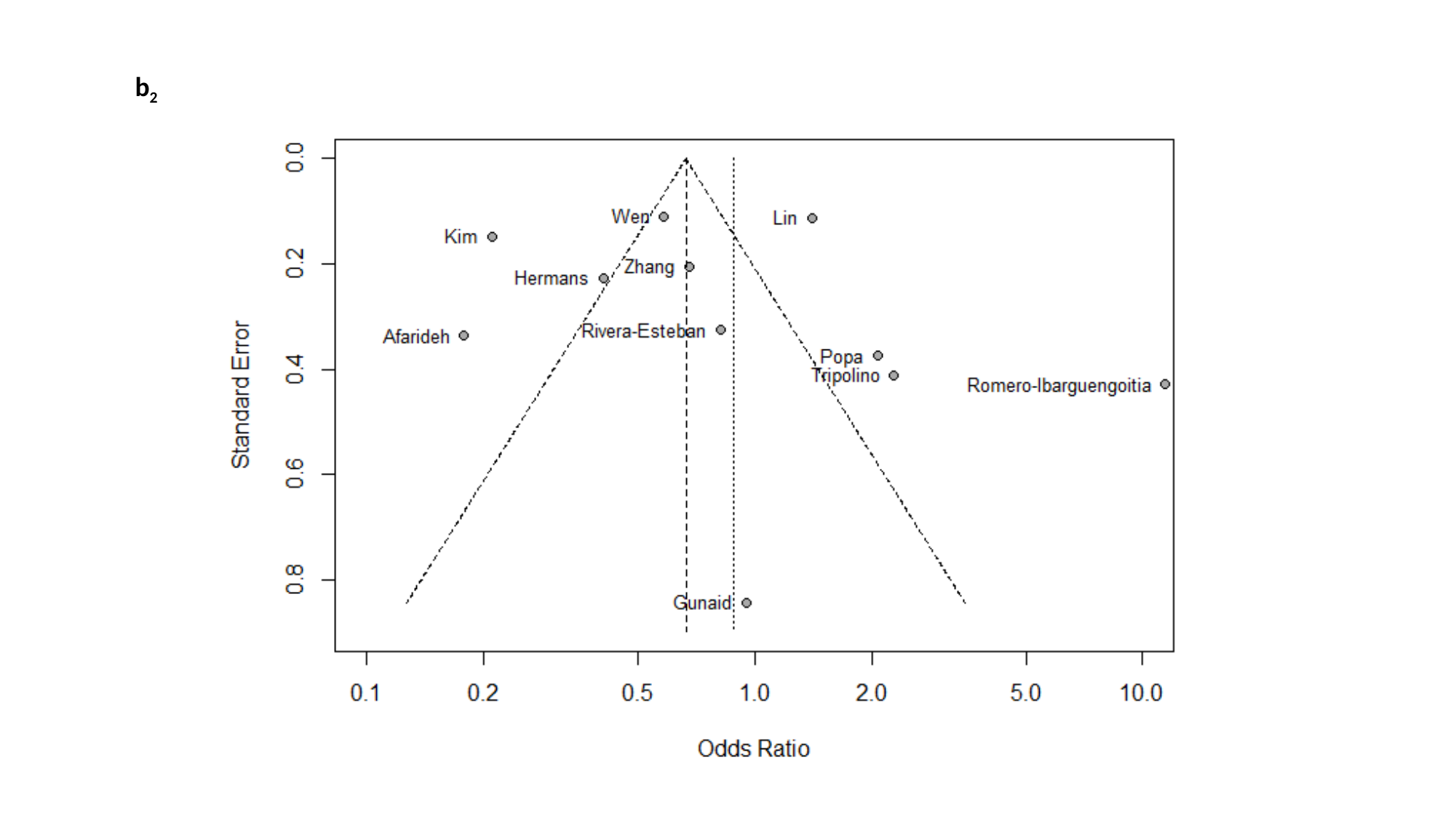

b2

## Slide 5
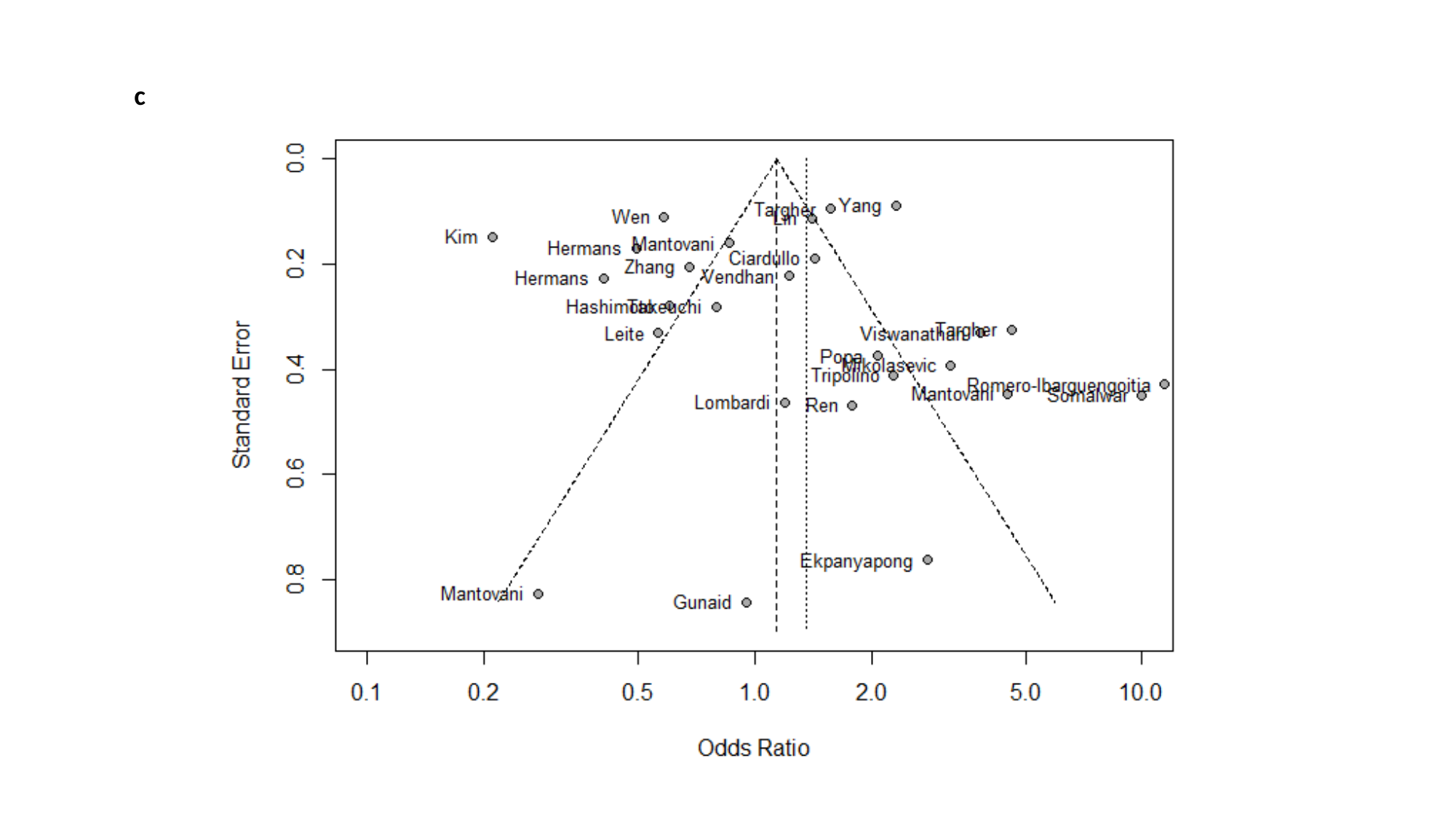

c

## Slide 6
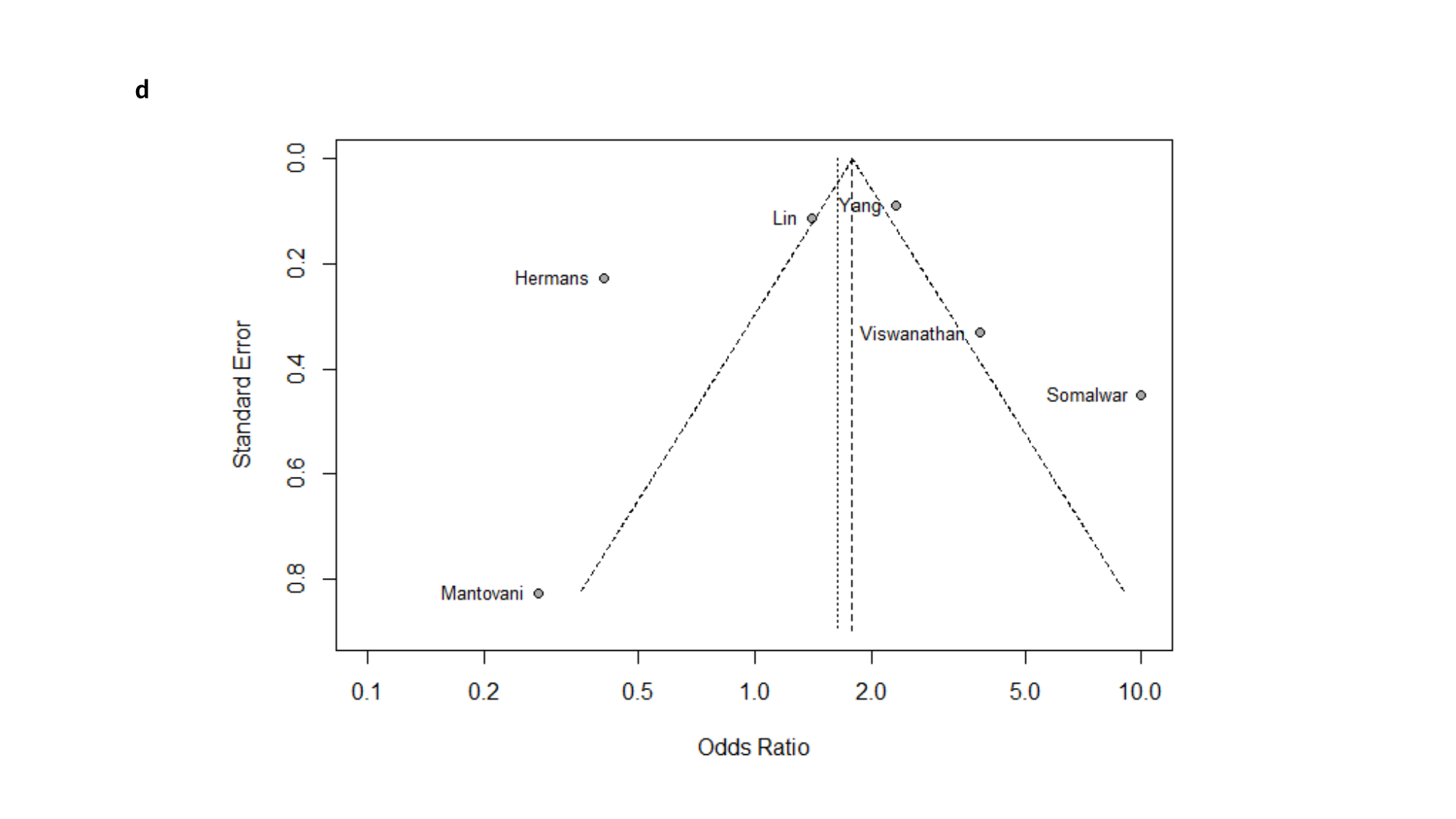

d

## Slide 7
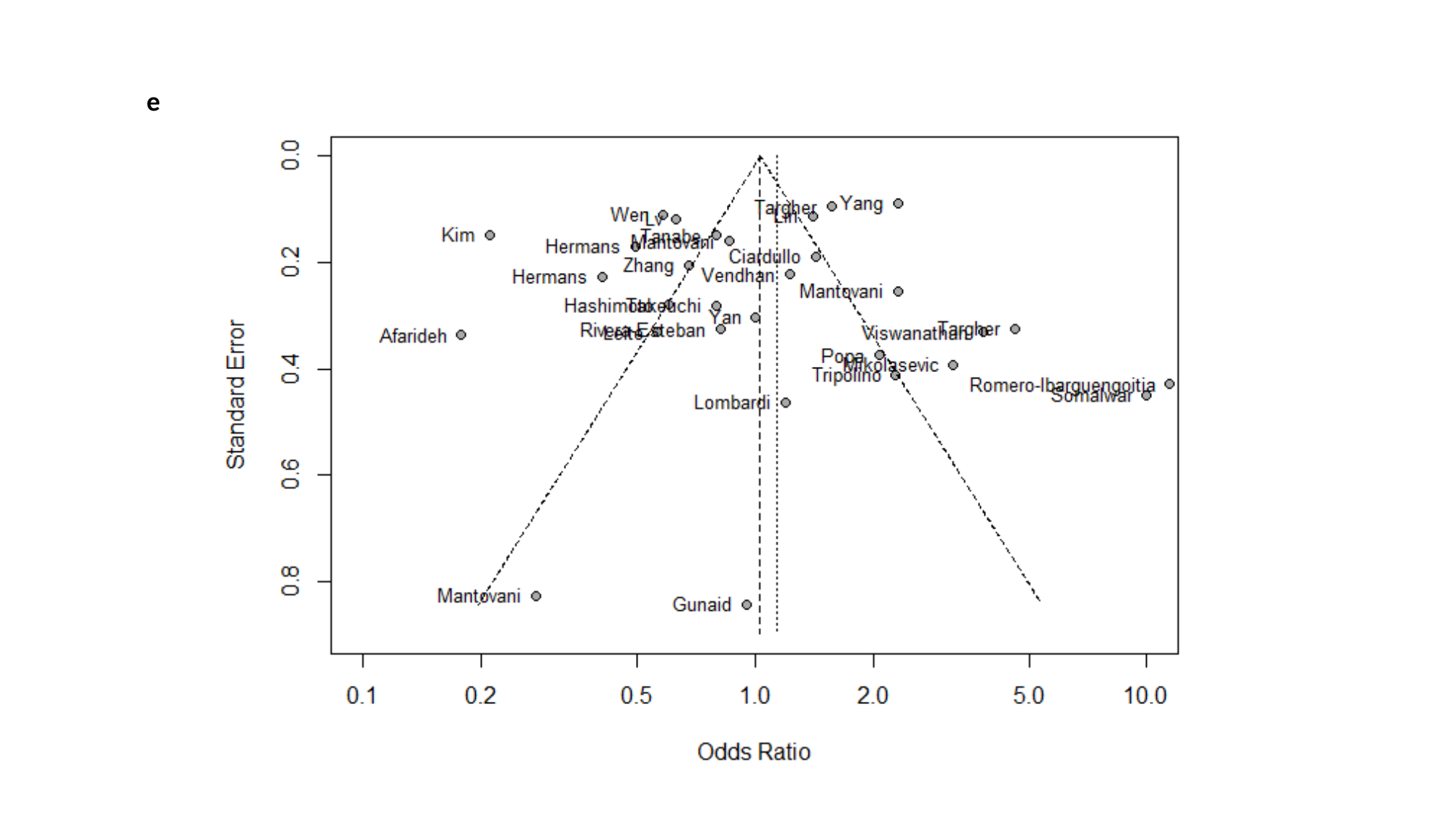

e

Supplement: Supplementary file 1 [file jpm-13-01148-s001.zip › supplementary figure S1.pptx]

## Slide 1
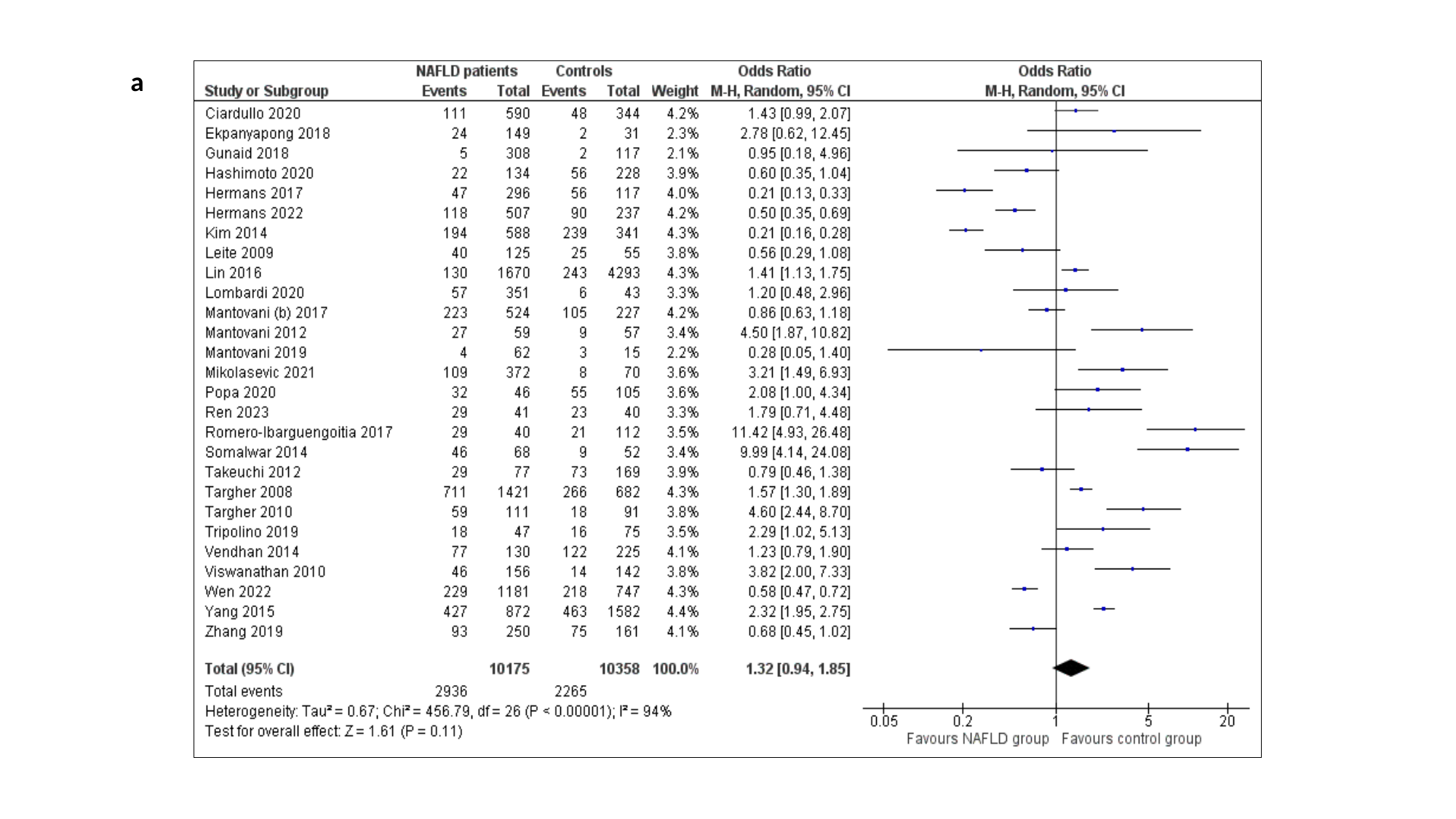

a

## Slide 2
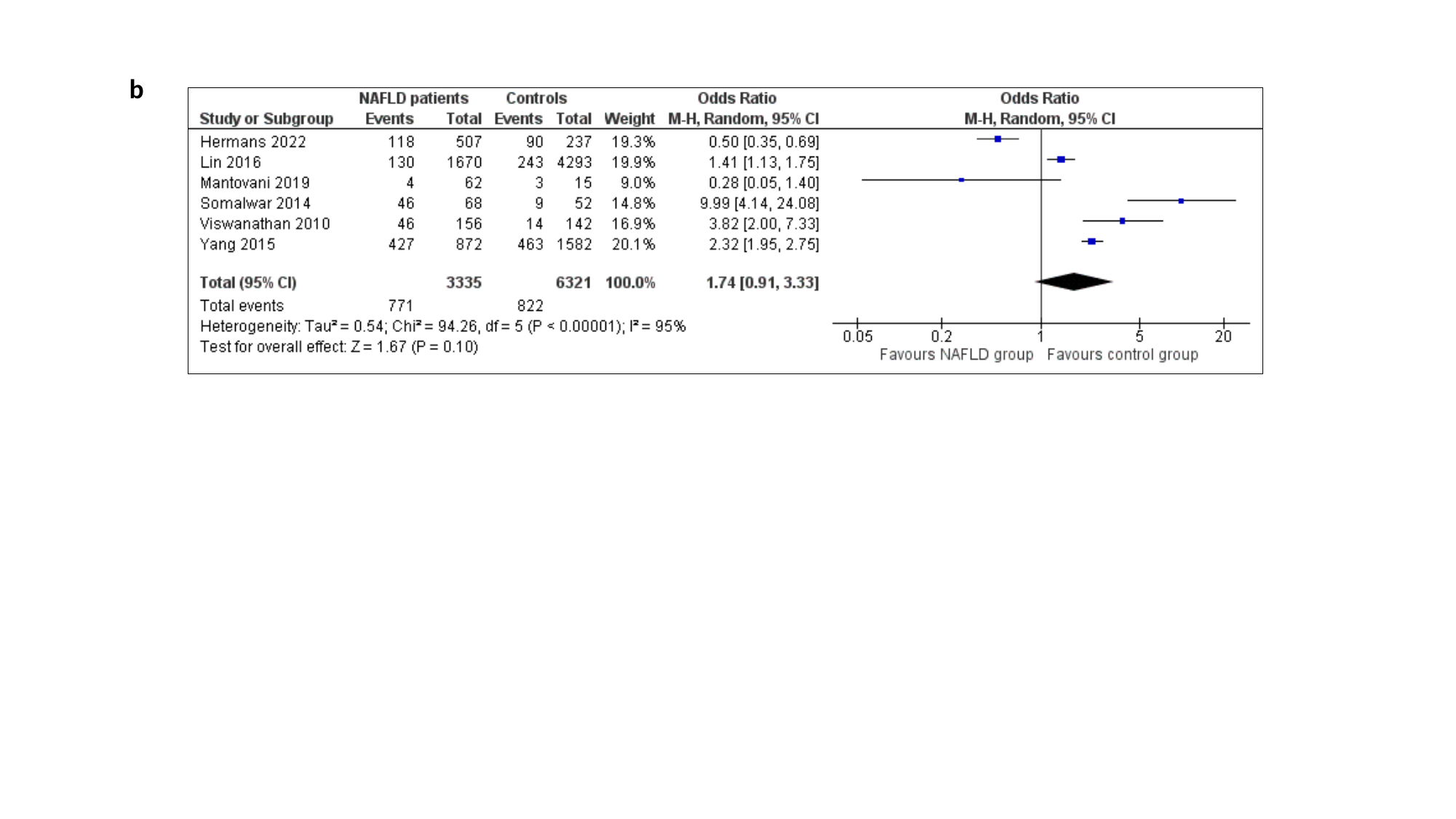

b

## Slide 3
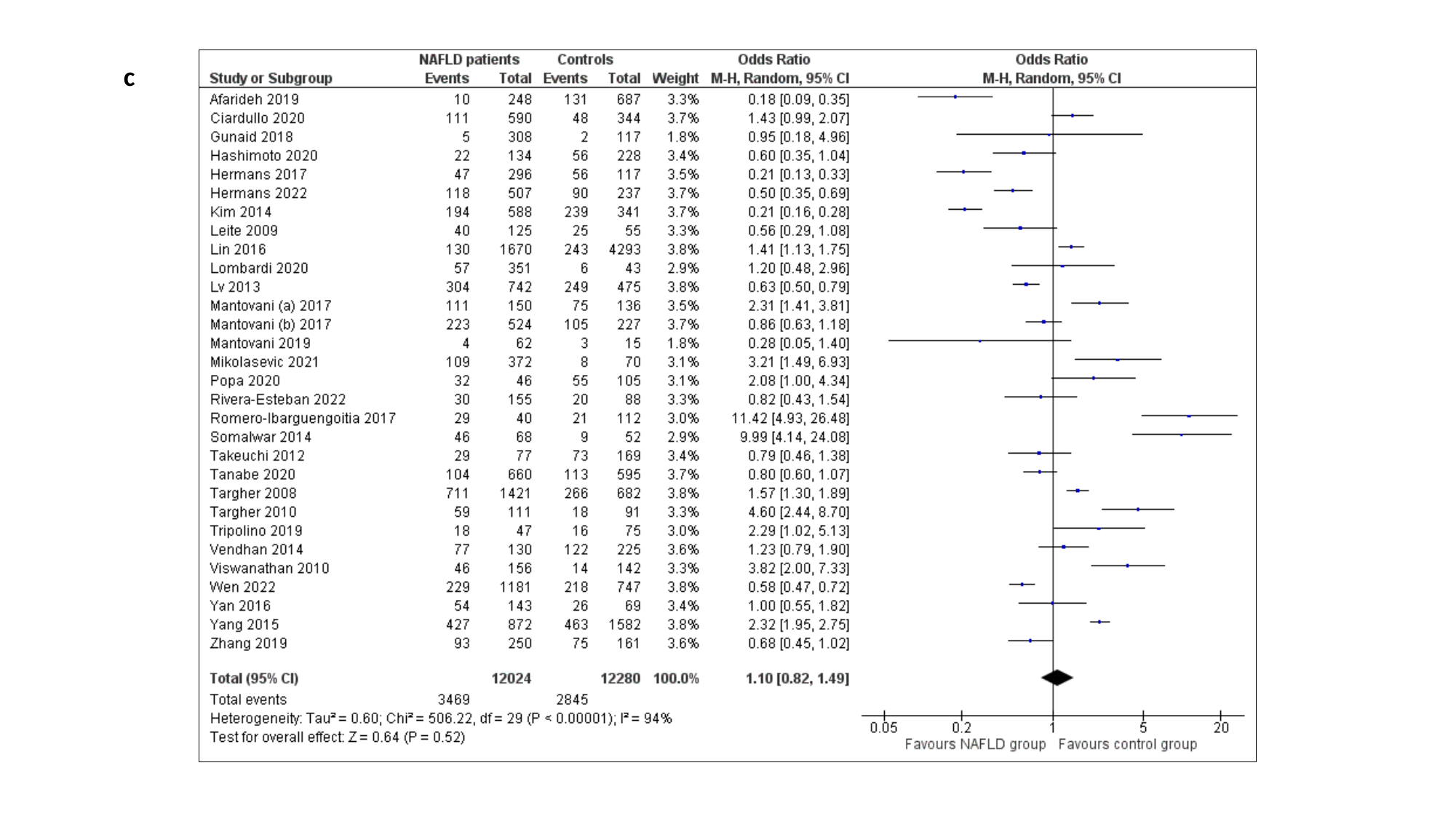

c

Supplement: Supplementary file 1 [file jpm-13-01148-s001.zip › supplementary figure S2.pptx]
